# Assessment of Mechanical Damage and Germinability in Flaxseeds Using Hyperspectral Imaging

**DOI:** 10.3390/foods13010120

**Published:** 2023-12-29

**Authors:** Mohammad Nadimi, L. G. Divyanth, Muhammad Mudassir Arif Chaudhry, Taranveer Singh, Georgia Loewen, Jitendra Paliwal

**Affiliations:** 1 Department of Biosystems Engineering, University of Manitoba, Winnipeg, MB R3T 5V6, Canada; 2 Center for Precision and Automated Agricultural Systems, Washington State University, Prosser, WA 99350, USA; divyanth.l.girija@wsu.edu

**Keywords:** flaxseeds, hyperspectral imaging, chemometrics, mechanical damage, oilseed quality

## Abstract

The high demand for flax as a nutritious edible oil source combined with increasingly restrictive import regulations for oilseeds mandates the exploration of novel quantity and quality assessment methods. One pervasive issue that compromises the viability of flaxseeds is the mechanical damage to the seeds during harvest and post-harvest handling. Currently, mechanical damage in flax is assessed via visual inspection, a time-consuming, subjective, and insufficiently precise process. This study explores the potential of hyperspectral imaging (HSI) combined with chemometrics as a novel, rapid, and non-destructive method to characterize mechanical damage in flaxseeds and assess how mechanical stresses impact the germination of seeds. Flaxseed samples at three different moisture contents (MCs) (6%, 8%, and 11.5%) were subjected to four levels of mechanical stresses (0 mJ (i.e., control), 2 mJ, 4 mJ, and 6 mJ), followed by germination tests. Herein, we acquired hyperspectral images across visible to near-infrared (Vis-NIR) (450–1100 nm) and short-wave infrared (SWIR) (1000–2500 nm) ranges and used principal component analysis (PCA) for data exploration. Subsequently, mean spectra from the samples were used to develop partial least squares-discriminant analysis (PLS-DA) models utilizing key wavelengths to classify flaxseeds based on the extent of mechanical damage. The models developed using Vis-NIR and SWIR wavelengths demonstrated promising performance, achieving precision and recall rates >85% and overall accuracies of 90.70% and 93.18%, respectively. Partial least squares regression (PLSR) models were developed to predict germinability, resulting in R^2^-values of 0.78 and 0.82 for Vis-NIR and SWIR ranges, respectively. The study showed that HSI could be a potential alternative to conventional methods for fast, non-destructive, and reliable assessment of mechanical damage in flaxseeds.

## 1. Introduction

Flaxseed (*Linum usitatissimum* L.) is a valuable oilseed crop, well known for its numerous nutritional and medicinal benefits [1,2,3]. The cultivation of this versatile crop spans the globe, driven by its ability to provide sustainable sources of protein, dietary fibre, and omega-3 fatty acids [2,4]. The market value of flaxseeds was 425.3 billion US dollars in 2021, and it is expected to expand at a compound yearly growth rate of 12.8% [5]. However, the quality and yield of flaxseed can be substantially affected by various factors, including environmental stresses, pathogens, pests, and mechanical damage [6]. Henceforth, mitigating quality and quantity losses across the flaxseed supply chain is essential in the face of burgeoning market demand.

Among the threats mentioned above, mechanical damage to seeds during harvesting and post-harvest processing is widespread and carries substantial implications for the quality and yield of many crops. Seeds subjected to damage may lose their viability, becoming increasingly prone to insect and fungal infestations, which degrade seed quality and reduce market value [7]. Given the robust demand for flaxseeds and the rigorous import regulations in place, extensive quality standards have been instituted to dictate precise grading criteria [8]. These regulations allow minimal tolerance for kernel damage, posing a grave concern for flaxseed growers who strive to curtail mechanical damage during harvesting and handling. As such, there is a necessity for a reliable tool capable of detecting and quantifying mechanical damage in flaxseeds at crucial points, including ports of entry, trade points, and grain elevators.

Several studies have investigated the effects of moisture content (MC) and impact stress on the breakage susceptibility of various crops, including wheat [9,10], cowpea [11], chickpea [12,13], green and red lentil seeds [14], and corn [15,16,17,18]. These experiments have proven that modifying the seed MC can reduce kernel damage during processing. However, the studies primarily focused on visual inspection of external surfaces, which is time-consuming, subjective, and limited to detecting apparent external damage. Additionally, external and internal damage in seeds may not always be highly correlated, making it challenging to assess mechanical damage comprehensively [19]. Another compounding factor in determining the quality of flaxseeds using manual inspection is their small size.

Recently, radiographic imaging has emerged as a desirable approach for evaluating the internal structure of agri-food products [20,21,22,23,24,25,26,27,28]. This technique has been employed to assess mechanical damage to various crops, including maize [29,30], rice [31], kidney bean [32], soybean [33], and notably flaxseeds [34]. Particularly in the case of flaxseed, the use of 2D X-ray imaging coupled with machine learning tools demonstrated high efficacy, achieving accuracies of up to 91% in differentiating mechanically damaged flaxseeds [34]. Despite their robust statistical reliability, radiographic imaging methods are hindered by low throughput and elevated system costs [7]. Consequently, the flaxseed industry highly values an efficient alternative to monitor seed quality.

This study aims to investigate an alternative method for evaluating mechanical damage in flaxseed using spectroscopy-based techniques. Spectroscopy has emerged as a promising tool for agri-food quality evaluation, as it can provide rapid and non-destructive information about crop properties [35,36]. Hyperspectral imaging (HSI) is a spectral information acquisition method that integrates the capabilities of conventional NIR spectroscopy and imaging by providing simultaneous spectral and spatial information about the object of interest. The technique has been widely used for the characterization of several grain properties, such as moisture content [37], diseases [38,39], protein content [40], starch content [41], foreign material [42], insect infestation [43,44], and sprout damage [45]. Despite the immense potential of HSI, relatively few studies have explored its applicability in estimating seed mechanical damage [46,47]. For instance, a classification model based on least squares-SVM (LS-SVM) was proposed, using wavelengths selected through principal component analysis (PCA)-based variable importance analysis to differentiate damaged wheat kernels from healthy ones [46]. However, no attempts have been reported to evaluate mechanical damage in flaxseeds using spectroscopic techniques.

The present research aims to fill this knowledge gap and provide new insights into evaluating mechanical damage in flaxseeds using spectral information extracted from HSI in the visible-near infrared (Vis-NIR) and short-wave infrared (SWIR) range. In addition, expanding on the analysis of seed damage, this study also aims to estimate the germinability of flaxseeds using spectral data derived from HSI. Determining germinability is essential to seed physiology, as it provides insight into the seed’s potential to sprout and grow successfully. However, traditional methods of determining germinability can be time consuming, typically taking at least seven days [48]. In contrast, spectroscopic techniques offer the advantage of being non-destructive and quick, providing instant feedback on the germination potential. Therefore, such an approach can benefit agriculture and seed industry stakeholders by expediting the process. The insights gained from this study will enrich ongoing research to minimize grain losses in the seed industry.

## 2. Materials and Method

### 2.1. Sample Preparation

**Figure 1:**
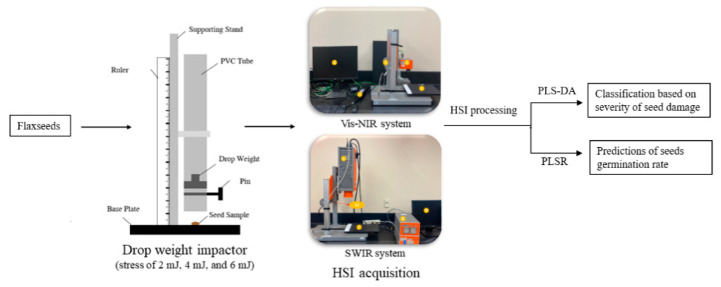
Methodology outlining the sample preparation and HSI spectra acquisition for seed-damage-based classification and germinability prediction.

The flaxseeds used in this study were obtained from a local producer in Arborg, Manitoba, Canada. The oilseeds were conditioned to three different moisture content (MC) levels, viz., 6%, 8%, and 11.5%, as determined by oven-drying at 103 °C for 72 h. The conditioned seeds were then subjected to impact testing using a drop weight impactor to apply mechanical forces at different energy levels of 2 mJ, 4 mJ, and 6 mJ (Figure 1). These impact energy (IE) levels were chosen based on preliminary testing to cause low, medium, and high levels of kernel damage, respectively. A set of control samples was also included for each MC level, where seeds were not subjected to any IE. In total, 3600 seeds (100 seeds × 4 impact energies × 3 MC levels × 3 replicates) were used in this study.

### 2.2. Germination Tests

The 3600 seeds were analysed as 144 sets of 25 each (144 × 25 = 3600) such that all the 25 seeds in a set experienced the same level of IE. Germination tests were performed following the guidelines of the Canadian Food Inspection Agency’s Canadian Methods and Procedures for Testing Seed [48]. Flaxseeds were placed on a grade 4 Whatman filter paper in a 9 cm Petri dish, followed by adding 5 mL of distilled water to perform germination tests. Hereinafter in this manuscript, a sample denotes the set of 25 seeds. The Petri dishes were placed inside a germination chamber (Home Herb Cultivator, Danby Inc., Guelph, ON, Canada) and germinated at 20 °C for 7 days. A seed was considered germinated when it exhibited a radicle length of at least 2 mm. The germinability in each sample set was determined using the equation below [49]:

(1) $$Germinability\;(\%)=\frac{N_{g}}{N_{t}}\times 100$$

where $N_{g}$ represents the number of germinated seeds at the end of the germination period, and $N_{t}$ denotes the total number of seeds used in the test.

### 2.3. Hyperspectral Imaging System and Image Acquisition

Hyperspectral images were acquired using line scanning systems operating in the Vis-NIR and SWIR ranges (SPECIM Spectral Imaging Ltd., Oulu, Finland). The Vis-NIR system operated in the wavelength range of 397.66 nm to 1003.81 nm, spanning 224 bands at a spatial resolution of 1024 × 896 pixels. On the other hand, the SWIR system produced 288 bands ranging from 953.36 nm to 2567.37 nm with a spatial resolution of 300 × 384 pixels. The exposure duration for Vis-NIR and SWIR was 20 ms and 8 ms, respectively.

To achieve thermal and temporal stability, both systems were switched on 30 min before data acquisition. The frame rate for each camera was set to 20 frames per second (FPS). The moving stage speed was set to 7 mm/s for the Vis-NIR and SWIR systems to gain the optimum frame rate and exposure duration aspect ratio. 

It is important to specify dark and white reference images for the system to estimate the relative reflectance image of each sample ($I_{relative}$). The dark reference image ($I_{dark}$) was obtained by closing the shutter of the camera, and the white reference image ($I_{white}$) was obtained with a 99% Spectralon (Labsphere, North Sutton, NH) matching reference standards. If $I_{sample}$ is the sample’s reflection image, then the relative reflection image of the sample was obtained following Equation (2). Similar to the germination test, the images were acquired in sets of 25 seeds. Hence, 144 samples were imaged using the Vis-NIR and SWIR wavelength ranges.

(2) $$I_{relative}=\frac{I_{sample}-I_{dark}}{I_{white-I_{dark}}}$$

### 2.4. Image Processing

**Figure 2:**
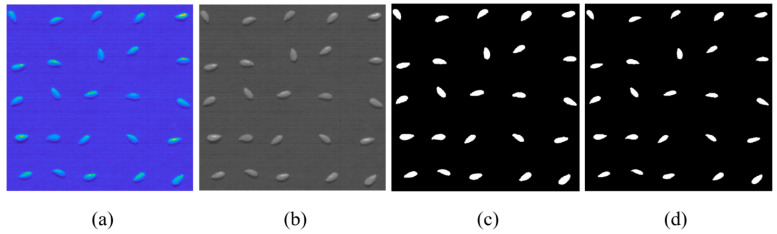
(**a**) Sample HSI of flaxseeds from the SWIR range, (**b**) raw image at a wavelength of 1287.94 nm, (**c**) ROI of the flaxseeds sample obtained after performing image thresholding, and (**d**) final processed ROI for calculating the mean spectra.

All image analyses were performed in MATLAB R2022a (The Mathworks Inc., Natick, MA, USA) software. The steps involved during image processing are illustrated in Figure 2. Image segmentation was performed using Otsu’s thresholding method [50] to capture the region of interest. A wavelength that offered better contrast between the pixels of flaxseed samples and the background was chosen, and the corresponding channel was used for image segmentation (Figure 2b). Subsequently, the threshold value was identified from the channels corresponding to 634.08 nm and 1287.94 nm for Vis-NIR and SWIR, respectively. Using the threshold value, a binary mask was created to segregate the pixels of interest (representing the flaxseeds) from the background (Figure 2c). A disk-shaped structuring element with a neighbourhood size of 3 × 3 pixels was used to erode the ROI (Figure 2d). This prevented the non-representative background pixels in the ROI’s boundary from contributing to the average spectrum. Finally, the mean spectra were obtained from this processed ROI by averaging the spectral reflectance values of all the representative pixels.

### 2.5. Spectral Dataset

The spectral data were investigated using principal component analysis (PCA), allowing for identifying and removing outliers and visualizing trends within the dataset. Occasional artifacts were observed in the reflectance curves of some samples obtained from the Vis-NIR system, likely resulting from non-uniform pixel intensities that arise from the manufacturing processes of InGaAs sensors. These erroneous spectra were duly excluded from the final dataset. Consequently, the spectral analysis utilized a dataset of 144 samples from the SWIR range and 141 samples from the Vis-NIR range (refer to Table 1).

To achieve a comprehensive prediction of seed damage severity, which directly impacts germinability, classification models were constructed using both the SWIR and Vis-NIR spectral ranges. These models aimed to categorize the seed damage into two broad groups of nil/low damage and medium/high damage, as suggested by previous studies using X-ray radiography [19] and the obtained PCA model observations. The nil/low damage class incorporated control samples and samples impacted at an IE of 2 mJ, while the medium/high damage class encompassed samples impacted with IEs of 4 mJ and 6 mJ. A detailed discussion of the differences in the microstructure of the above samples was provided elsewhere [19,34].

Before the development of the classification model, the dataset was split into calibration/training and test sets using the Kennard–Stone algorithm (KSA) [51]. The KSA maintains the identical distribution of the samples in the calibration and test sets and considers the data points along the dataset boundary [52]. Using the KSA, about 70% of the dataset was used as the training set (100 samples of SWIR and 98 of Vis-NIR range), and the remaining 30% was reserved for testing (44 samples of SWIR and 43 samples of Vis-NIR range). 

Recognizing the critical role of cross-validation in model evaluation, particularly with high-dimensional data characteristic of HSI, each calibration model employed a Venetian blinds cross-validation method featuring 10 data splits. This technique involves dividing the dataset into ‘k’ subsets to ensure a comprehensive evaluation. During each cross-validation cycle, a different subset is selected as the test set, while the rest serve as the training set [53]. Such a methodology not only enhances the robustness of the model but also guarantees that every data point contributes to the validation process, thereby bolstering the overall reliability of the findings.

### 2.6. Data Mining Techniques and Spectral Analysis

Principal component analysis (PCA) is a method for transforming data, condensing a high number of interrelated variables into a smaller set of orthogonal variables known as principal components (PCs), which are uncorrelated [54,55]. Due to the high dimensionality of spectral data, dimensional reduction and visualization were performed using PCA (using the PLS toolbox (Eigenvector Research, Inc., Manson, WA, USA)) to distinguish flaxseed samples based on their IE levels and groups. Since the initial PCs represent the majority of the variance of the dataset, inspecting the dataset using these components in the transformed feature space can provide a valuable conjecture for classification. Before the transformation, the SWIR and Vis-NIR datasets were mean-centred and processed using the standard normal variate (SNV) transformation to reduce the scattering effect and intra-spectral correlation [56]. 

Similar to PCA, PLS is also a linear transformations-based dimensionality reduction technique used to classify, differentiate, predict, or show correlations between groups of examined variables by using a smaller number of latent variables [57]. For the development of prediction models, the PLS technique is better than PCA because of its supervised approach, especially in the case of a specific set of independent variables. The primary goal of PLS is to extract a group of factors or latent variables from a dataset and develop a linear model such that:

(3) $$Y=T_{k}C_{k}+E_{k}=X{W_{k}}^{*}C_{k}+E_{k}$$

where Y and X are the response variable and predictor variable set matrices, respectively, $T_{k}$, $C_{k}$, ${W_{k}}^{*}$ are the PLS transformation matrices, and $E_{k}$ denotes the residuals matrix. 

Considering the high dimensionality of spectral data, it becomes imperative to identify and select the most significant wavelengths with representative features to expedite the prediction of damage classes. Leveraging wavelength selection when calibrating prediction models not only reduces computational complexity but also aids in designing portable sensors. Consequently, we utilized the partial least squares-variable importance in projection (PLS-VIP) technique to discern important wavelengths and facilitate the construction of prediction models [58,59]. This approach gauges the importance of each wavelength based on its contribution to the variance explanation of each PLS component [58]. In addition to the model outcomes derived from selected wavelengths, which are discussed in detail in Section 3, an assessment of prediction performance using the entire spectrum of wavelength ranges has been provided in the supplementary materiall.

After the selection of important wavelengths, the PLS-discriminant analysis (PLS-DA) supervised classification algorithm [60,61] was implemented to classify flaxseeds based on the induced seed damage. Two models were developed using the critical wavelengths observed in the SWIR and Vis-NIR ranges, respectively. The models were evaluated using precision, recall, classification accuracy, and F1-score metrics in the calibration, cross-validation, and test datasets (Equations (4)–(7)). Precision value for a class is defined as the ratio of true positives (TP) to the total number of objects predicted for this class (TP + false positives (FP)), while recall rate is the ratio of TP to the actual number of objects in that class (TP + false negatives (FN)). The F1-score is the harmonic mean of precision and recall.

(4) $$Precision=\frac{TP}{TP+FP}$$

(5) $$Recall=\frac{TP}{TP+FN}$$

(6) $$F1-score=\frac{2\times Precision\times Recall}{Precision+Recall}$$

(7) $$Accuracy=\frac{TP+TN}{TP+TN+FP+FN}$$

Furthermore, PLS regression (PLSR) models were developed to predict the germinability of flaxseeds based on the reflectance spectral data. The *plsregress* function of MATLAB was used to execute PLSR via the SIMPLS algorithm [62]. The performance of the PLSR model was examined using the coefficient of determination (R^2^) and root mean square error (RMSE) metrics. 

## 3. Results and Discussion

**Figure 3:**
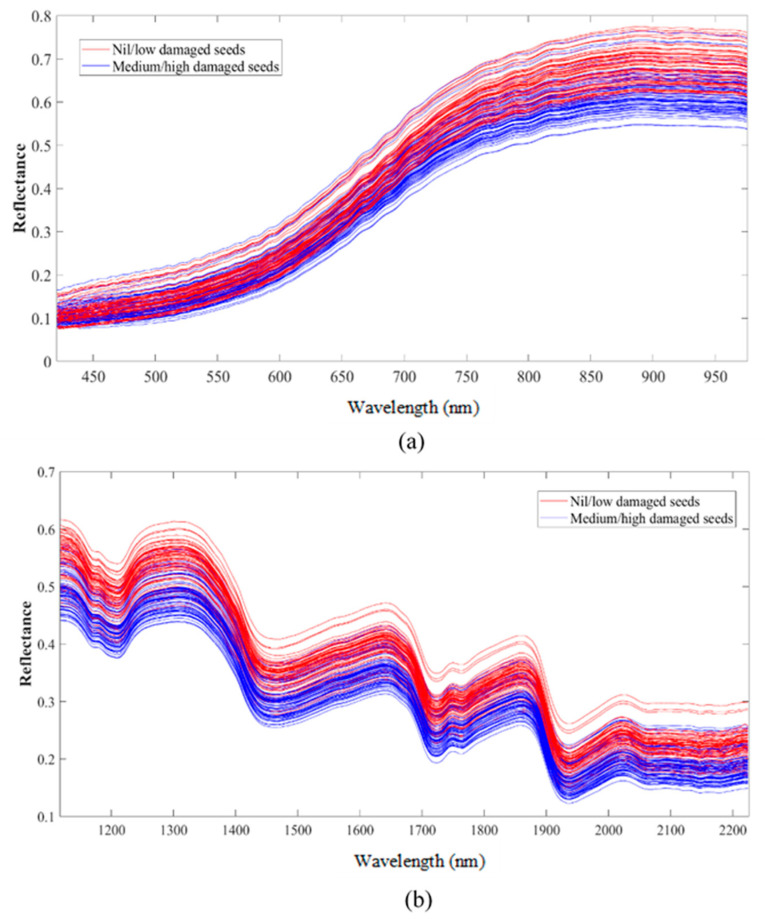
Raw spectra of the flaxseed samples in the (**a**) Vis-NIR range, and (**b**) SWIR range.

The raw spectra of the flaxseeds in the Vis-NIR and SWIR ranges can be seen in Figure 3a,b, respectively. A close separation can be noted among the spectra corresponding to the two groups of seed damage in both wavelength ranges. However, the spectral signatures and curves were similar throughout the entire wavelength range. In the Vis-NIR region, the distinction of the samples’ spectra of the two groups is predominant in the higher wavelengths (>800 nm). Moreover, the colour features of the seeds are represented in the Vis-NIR spectra by wavelengths in the range of 650–750 nm. The peaks in the second and first overtone regions (~1450 and 1950 nm) in the SWIR range can be associated with the moisture content of the samples. The reflectance in wavelength bands of 1750–2000 nm, 1600–1800 nm, and 1250–1350 nm can be related to the first overtone of fatty acids, the presence of carbohydrates, and proteins, respectively [63].

### 3.1. Germination Test Results

**Figure 4:**
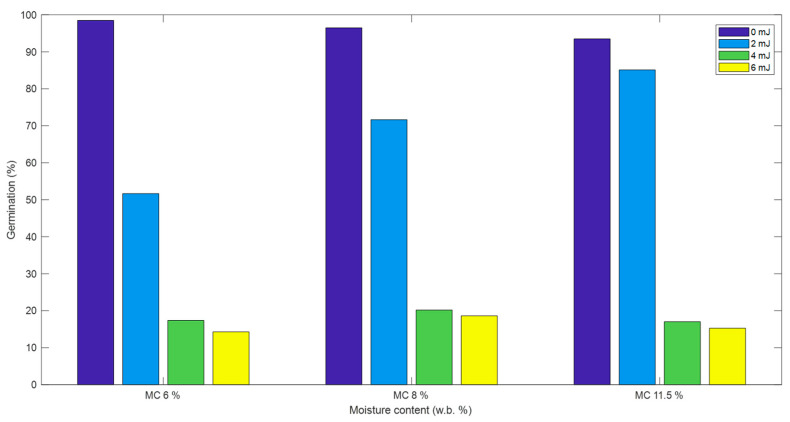
Effect of moisture content and impact energy (IE) on the germinability of flaxseeds (adapted from [19], with permission from Elsevier, 2023).

The effect of mechanical damage on flaxseed germination is shown in Figure 4. It is apparent that the germinability of flaxseeds decreased as their IE increased from 0 mJ (control) to 2 mJ and 4 or 6 mJ (the germination rate was almost similar for samples impacted with 4 mJ and 6 mJ), at all moisture contents. The effect of moisture content on germinability was minimal for high-IE compared to low-IE. When impacted with an energy of 2 mJ, the germinability was around 56% and 85% for seeds at 6% and 11.5% MC, respectively. A drastic decline in germinability was observed when the IE was increased to 4 mJ. However, there was no remarkable difference between the germinability of seeds impacted with 4 mJ and 6 mJ IE. 

### 3.2. PCA-Based Data Exploration

PCA was performed on the mean-centred spectral reflectance of flaxseed samples to understand the classification potential of Vis-NIR and SWIR wavelengths. Based on the PCA results, it can be inferred that PCA models in the Vis-NIR range can distinguish samples based on the two respective classes. It can also be concluded that the PCA model in the SWIR range was more elaborate, where PC2 depicted the grouping between designated classes and PC1 showed groupings between the nil/low damaged seeds class. This observation suggests a more nuanced and comprehensive characterization of the data in the SWIR range, emphasizing the significance of the SWIR spectral region in capturing meaningful variations related to mechanical damage in the flaxseeds.

**Figure 5:**
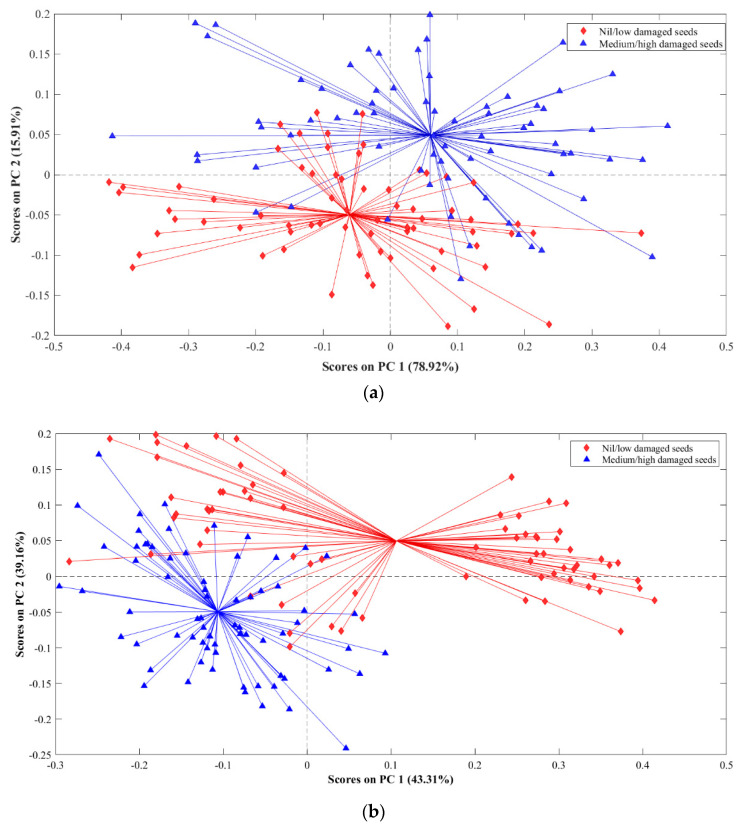
Scores plot for classifying flaxseeds into two broad groups based on their impact energy using the reflectance spectra; (**a**) Vis-NIR, and (**b**) SWIR wavelengths.

The first two principal components (PCs) were extracted for visualization purposes (Figure 5), which covered about 95% of the total variance of the Vis-NIR dataset (Figure 5a) and 82% in case of the SWIR dataset (Figure 5b). PCA model for the Vis-NIR range showed that PC2 could distinguish between the two classes of flaxseeds. The scores plot for the SWIR dataset depicts that PC1 is responsible for distinguishing the two classes. However, a clear grouping was observed within the nil/low damaged seeds class along the PC1 axis. This grouping can be associated with the control samples and low-damaged samples, respectively. Overall, the satisfactory results of PCA for group-based classification of flaxseeds based on IE, especially using the Vis-NIR range wavelengths, encouraged the development of supervised classification models based on PLS-DA. 

### 3.3. Wavelength Selection and PLS-DA-Based Classification

**Figure 6:**
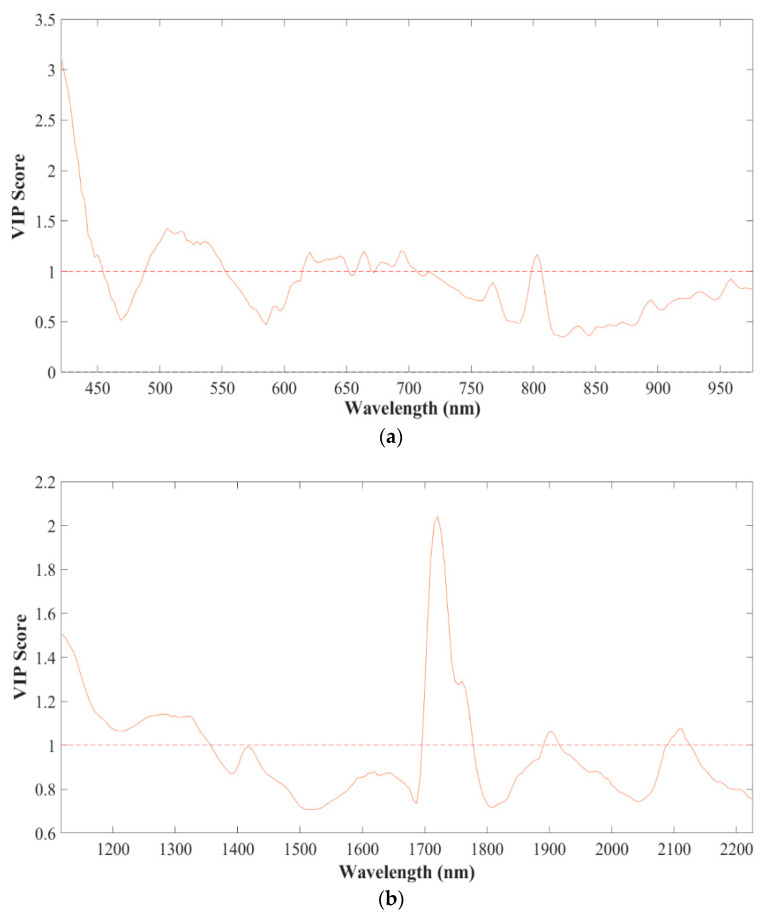
Wavelength importance scores based on PLS-VIP for (**a**) Vis-NIR and (**b**) SWIR wavelength ranges.

PLS-VIP analysis was performed to select the effective wavelengths from the full spectral region of the Vis-NIR and SWIR ranges (Figure 6). A high VIP score for a wavelength underscores its high importance for the prediction model. Subsequently, a VIP score greater than 1.00 was considered the variable/wavelength selection criterion. In the Vis-NIR data, wavelengths in the 485–550 nm, 620–700 nm, and 805–815 nm ranges yielded high VIP scores. On the other hand, wavelengths in the range 1700–1780 nm, 1890–1920 nm, and 2080–2130 nm exhibited high VIP scores in the SWIR data. 

Due to flaxseeds’ high unsaturated fatty acid content, they are prone to oxidative damage during processing. The mechanical damage imparted to the seeds in our experiments compromised their physical integrity, exposing their internal constituents to oxygen. In Figure 6a, the peaks observed for the VIP scores are associated with the colour changes in flaxseeds attributable to the oxidation of polyunsaturated fatty acids (PUFAs) such as ALA and phenolic compounds. On the other hand, for the SWIR range, a notable peak was observed between 1700 and 1800 nm, which is associated with proteins. This is because mechanical damage leads to the denaturation and fragmentation of proteins, which indirectly contribute to the oxidation by releasing enzymes such as lipases and lipoxygenases. These enzymes, in turn, generate reactive oxygen species (ROS) which further promote oxidative reactions [63,64,65,66].

Subsequently, PLS-DA models were developed for supervised classification based on the identified subset of effective wavelengths. The classification confusion statistics obtained using the Vis-NIR range are shown in Table 2. The training set achieved an accuracy of 89.80%, with 91.49% recall for the nil/low-damaged class and a precision of 91.84% for the medium-/high-damaged flaxseeds class. Moreover, a five-fold cross-validation on the calibration set showed an accuracy of 88.77%. Classification performance on a spectral dataset of 43 external test samples was evaluated to determine the reliability of the calibration model. Satisfactory classification performance was observed on the test set, with an accuracy of 90.70% and precision and recall rates > 85% for both classes. Since most of the wavelengths used for this classification model are from the 480–720 nm range, it can be deduced that the prediction was closely associated with the colour features of the seeds. It is evident that damaged seeds exhibit different colour characteristics in the impaired region, affecting the spectral response. In the case of full-wavelength data analysis, an accuracy of 88.37% was achieved for the test set under the Vis-NIR range (Table S1 (supplementary files)).

On the other hand, another PLS-DA model was developed using the selected wavelengths from the SWIR range. Table 3 depicts the confusion matrices in the training set, cross-validation, and test set to classify induced mechanical damage to flaxseeds. The model showcased 90.00% and 89.00% accuracy during calibration and five-fold cross-validation. On the test set of 44 samples, the classification accuracy was 93.18%, slightly better than the model developed from the Vis-NIR wavelength range. An increase in the recall and precision values for medium-/high-damaged and nil/low-damaged classes was observed, respectively. Therefore, the selected wavelengths from the SWIR range accorded a relatively better classification model than their Vis-NIR counterparts. In the case of full-wavelength data analysis, an accuracy of 93.18% was achieved for the test set under the Vis-NIR range (Table S2 (supplementary files)).

In light of these findings, it is important to consider the cost-effectiveness of Vis-NIR technology compared to the slightly superior performance of the SWIR range. Although SWIR exhibited marginally better results, the substantial cost advantages associated with Vis-NIR technology cannot be overlooked. For instance, Vis-NIR detectors commonly employ Silicon (Si) detectors, which are considerably more cost effective than those used in SWIR technology. As such, despite the minor trade-off in performance, Vis-NIR presents a more economically viable solution for a broader range of applications.

Additionally, the effectiveness of wavelength selection in maintaining the system’s performance is noteworthy. We effectively reduced data complexity by narrowing the spectrum to key wavelengths without compromising accuracy. This reduction in complexity translates into lower computational demands, leading to quicker processing times and decreasing operational costs.

Furthermore, these results highlight the potential for developing multi-wavelength systems as alternatives to hyperspectral ones. By strategically selecting wavelengths, such systems can maintain comparable performance while remarkably reducing data storage and processing power requirements. This minimizes system complexity and cost, enhancing the feasibility of spectral analysis applications. The study illuminates the importance of balancing system performance, cost, and complexity, paving the way for optimized and economical spectral analysis technologies.

It is also worth mentioning that in the landscape of techniques employed for mechanical damage evaluation in crops, traditional methods predominantly involve subjective visual assessments by technicians. While widely used, this approach often samples only a small fraction of the inbound crop and is prone to human error. Recent advancements have led to the exploration of X-ray-based methods for automated mechanical damage assessment in flaxseeds, where classification accuracies for nil/low versus medium/high damage seeds were reported as 87.2% and 95.2%, using SVM and CNN models, respectively [9,34]. However, X-ray based methods typically face challenges in throughput, limiting their practicality in large-scale operations. In contrast, the present work demonstrated the potential of HSI as a robust alternative. Unlike single-point spectroscopy techniques, HSI offers the unique advantage of both spatial and spectral evaluation, enabling a more comprehensive analysis of crop quality. HSI has already shown promise in assessing various crop properties, including compositional, functional, and sanitary indicators [7]. Our research extends this utility to the evaluation of mechanical damage in crops, and therefore, contributes to the growing body of evidence supporting the versatility and efficiency of HSI in agri-food product quality assessment.

### 3.4. Prediction of Germinability

For the prediction of germinability, several data pre-processing methods were employed similar to the classification process. These encompassed techniques viz., mean centring, standard normal variate (SNV), as well as a combination of mean centring and Savitzky–Golay 1st derivative. The most effective calibration performance was achieved using mean centring only.

Two PLSR models, one for each spectral range, were developed to predict the germinability of seeds. The Vis-NIR model yielded an R^2^-value of 75.62% for calibration and 72.8% for the cross-validation set. Utilizing five latent variables (LVs), the model produced a root mean square error of calibration (RMSEC) of 19.26% and a root mean square error of cross-validation (RMSECV) of 20.36%. The first LV (LV1) accounted for 95.49% of the data’s covariance. An external prediction dataset of samples was utilized to assess the calibration model’s reliability. The external prediction produced an R^2^ of 78.37% and a root mean square error of prediction (RMSEP) of 22.08%.

**Figure 7:**
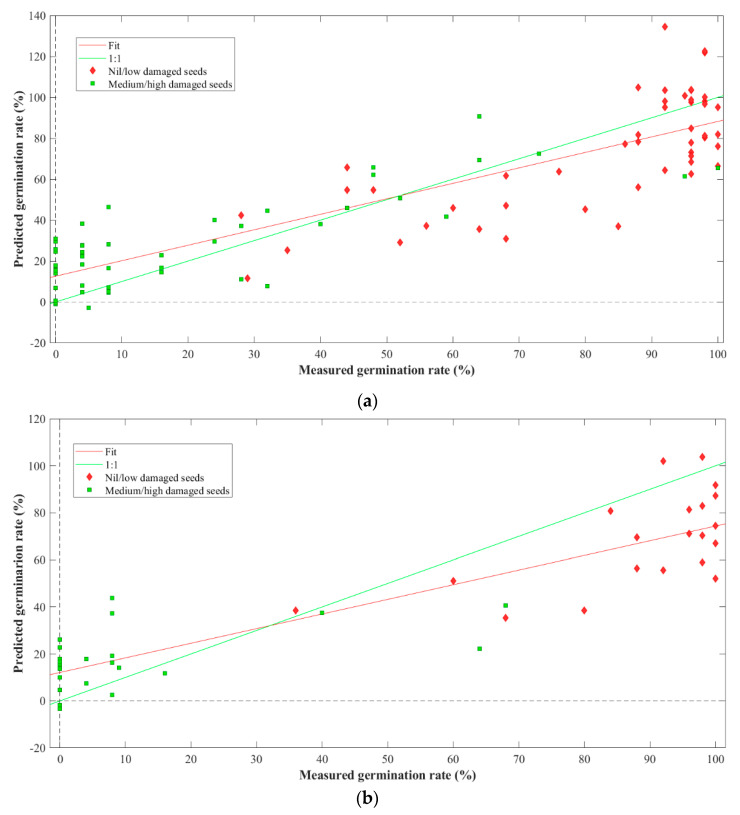
Regression plots for the prediction of germinability of flaxseeds using Vis-NIR HSI (**a**) cross-validation and (**b**) testing.

**Figure 8:**
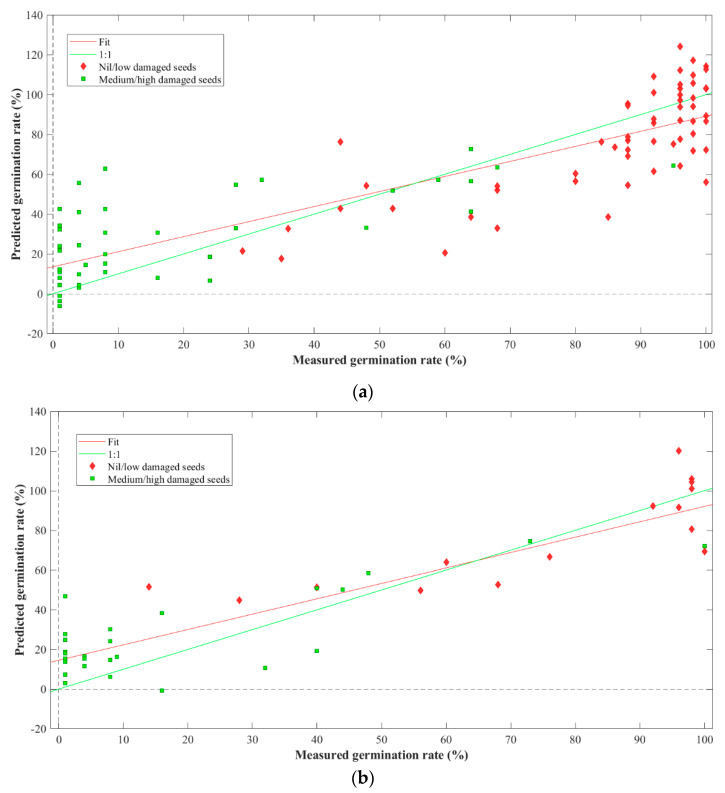
Regression plots for the prediction of germinability of flaxseeds using SWIR HSI (**a**) cross-validation and (**b**) testing.

The second model was employed using the SWIR ranges. The model yielded an R^2^ value of 76.22% for calibration and 74.47% for cross-validation. The model resulted in an RMSEC of 19.17% and an RMSECV of 19.87%. Similarly, the first LV described 98.33% covariance of the data. The external prediction generated an R^2^ of 82.15% and an RMSEP of 17.23%. Figure 7 and Figure 8 illustrate the regression plots for cross-validation and prediction of the germinability of different groups of flaxseeds in the Vis-NIR and SWIR ranges, respectively.

While the predictive models developed for the germinability of the mechanically damaged seeds demonstrate encouraging performance, pinpointing the exact germinability remains challenging, as reflected in Figure 7 and Figure 8. This difficulty could stem from the inherent limitation of the employed HSI regarding the shallow penetration depth of the signal. Nevertheless, it is crucial to recognize HSI’s invaluable role in extracting critical data related to mechanical damage to seeds. HSI application proves itself an efficient tool for the preliminary screening of flaxseeds, delivering quick, non-destructive assessments. This underscores the technology’s potential to substantially contribute to the seed industry.

## 4. Future Work

Future directions for this research are plentiful and exciting, particularly as HSI technology and data processing techniques continue to advance. Despite the promising results from this study’s Vis-NIR and SWIR spectral ranges, there is considerable scope for refinement. Innovative imaging techniques with cutting-edge camera technology could enhance the resolution and sensitivity of spectral images, thereby generating richer datasets for analysis.

With the increasing capabilities of machine learning and artificial intelligence, data processing and analysis techniques can be refined and improved. While the pre-processing methods used in this study were chosen for their demonstrated efficacy in similar research contexts, exploring a range of HSI pre-processing techniques remains an important avenue for future work. Such systematic studies could provide valuable insights into optimizing data processing of HSI hypercubes for crop quality monitoring. 

Future research could also explore using decision trees or other interpretable models in the context of HSI for agri-food quality analysis. This would provide an interesting comparison to the chemometric methods employed in this study and could enhance the overall understanding of HSI applications in agriculture. Furthermore, deeper learning models, such as convolutional neural networks (CNNs), could be instrumental in managing the high dimensionality of hyperspectral data, potentially surpassing existing models in classification and regression tasks.

In the future, a multi-modal approach that combines HSI with other non-destructive testing methods, such as X-ray or thermal imaging, could be adopted. This comprehensive approach could provide a more detailed and holistic characterization of seed quality, contributing to a broader understanding of seed physiology. Longitudinal studies could provide impactful benefits to this area of research, tracking the spectral changes in seeds over time under various storage and environmental conditions. This could enhance understanding of how these conditions impact seed quality and potentially improve the accuracy and robustness of the predictive models.

While this study focused on flaxseeds, the methods and findings could be applied to other types of seeds. Future research could extend the use of HSI in assessing the quality and germinability of various crop seeds, possibly contributing to the broader goals of food security and sustainable agriculture. In the context of germinability, this study examined performance in predicting the germination of mechanically damaged seeds. Extending this prediction to sound and poorly stored damaged seeds could offer new insights.

In summary, the future of HSI in seed quality analysis is promising. Continued research in this domain will undoubtedly lead to remarkable advancements in the agricultural and seed industries.

## 5. Conclusions

This research has effectively demonstrated the feasibility and applicability of hyperspectral imaging (HSI) as a non-destructive and rapid technique in seed quality assessment. Through the classification of flaxseeds based on mechanically induced damage and the prediction of their germinability, the potential of HSI technology to provide insightful data in a non-invasive manner has been highlighted. The comprehensive analysis conducted in this study has revealed the inherent value present in both the visible-near infrared (Vis-NIR) and the short-wave infrared (SWIR) ranges of HSI. These spectral ranges offer considerable detail regarding the characteristics of flaxseeds and the extent of their mechanical damage. While the SWIR range displayed marginally superior classification accuracy and prediction capability, both spectral ranges exhibited substantial success in identifying varying damage levels in flaxseeds. The results of this research not only attest to the merits of HSI as a tool for seed quality evaluation, but also highlight the potential for further optimization and expansion of its applications. The SWIR range, with its slightly better performance, does signal the possibility of refining the technique for increased accuracy and reliability. Indeed, the slight advantage of SWIR should not overshadow the capabilities of Vis-NIR. Vis-NIR technology often entails lower costs, primarily due to inexpensive optical components, so it represents a cost-effective solution for most applications. Furthermore, as noted in the study, the selection of wavelengths did not compromise system performance despite simplifying the data. This carries substantial advantages, such as decreased computational demands, leading to cost and time efficiencies. In conclusion, this study forms a robust foundation for future research, setting the stage for refining the application of HSI for flaxseed assessment and extending its potential to other seed varieties. 

## Figures and Tables

**Table 1:** Summary of the flaxseeds’ spectral dataset used for the study.

Wavelength Range	Group	Impact Energy	Number of Samples
Vis-NIR	Nil/low damaged	0 mJ (control samples)	36
		2 mJ	34
	Medium/high damaged	4 mJ	35
		6 mJ	36
SWIR	Nil/low damaged	0 mJ (control samples)	36
		2 mJ	36
	Medium/high damaged	4 mJ	36
		6 mJ	36

**Table 2:** PLS-DA results for mechanical damage classification using Vis-NIR wavelength range.

**Calibration**				
	True class			Recall (%)
Predicted class		Nil/low	Medium/high	
	Nil/low	43	4	91.49
	Medium/high	6	45	88.24
Precision (%)		87.76	91.84	
**Cross-validation**				
	True class			Recall (%)
Predicted class		Nil/low	Medium/high	
	Nil/low	43	5	89.58
	Medium/high	6	44	88.00
Precision (%)		87.76	89.80	
**Test**				
	True class			Recall (%)
Predicted class		Nil/low	Medium/high	
	Nil/low	18	1	94.74
	Medium/high	3	21	87.50
Precision (%)		85.71	95.45	

**Table 3:** PLS-DA results for mechanical damage classification using SWIR wavelength range.

**Calibration**				
	True class			Recall (%)
Predicted class		Nil/low	Medium/high	
	Nil/low	50	6	89.29
	Medium/high	4	40	90.91
Precision (%)		92.59	86.96	
**Cross-validation**				
	True class			Recall (%)
Predicted class		Nil/low	Medium/high	
	Nil/low	50	7	87.72
	Medium/high	4	39	90.70
Precision (%)		92.59	84.78	
**Test**				
	True class			Recall (%)
Predicted class		Nil/low	Medium/high	
	Nil/low	17	2	89.47
	Medium/high	1	24	96.00
Precision (%)		94.44	92.31	

## Data Availability

Data are contained within the article and supplementary materials.

## Supplementary Materials

The following supporting information can be downloaded at: https://www.mdpi.com/article/10.3390/foods13010120/s1. Table S1. PLS-DA results for mechanical damage classification using Vis-NIR wavelength range (without wavelength selection). Table S2. PLS-DA results for mechanical damage classification using SWIR wavelength range (without wavelength selection).
