# Supplementary material for: Assessment of Mechanical Damage and Germinability in Flaxseeds Using Hyperspectral Imaging
Source: Foods. 2023 Dec 29;13(1):120. doi: 10.3390/foods13010120 (PMC10778999; doi:10.3390/foods13010120)
Supplement: Supplementary file 1 [file foods-13-00120-s001.zip › foods-2766187-supplementary.pdf]

Supplementary Material

Assessment of Mechanical Damage and Germinability in Flaxseeds Using Hyperspectral Imaging

Mohammad Nadimi [1], L. G. Divyanth [2], Muhammad Mudassir Arif Chaudhry [1], Taranveer Singh [1], Georgia Loewen [1] and Jitendra Paliwal [1,*]

1 Department of Biosystems Engineering, University of Manitoba, Winnipeg, MB R3T 5V6, Canada; nadimim@myumanitoba.ca (M.N.); mudassir.chaudhry@umanitoba.ca (M.M.A.C.); singht9@myumanitoba.ca (T.S.); georgiabelle@hotmail.ca (G.L.)
2 Center for Precision and Automated Agricultural Systems, Washington State University, Prosser, WA 99350, USA; divyanth.l.girija@wsu.edu (L.G.D.);
* Correspondence: j.paliwal@umanitoba.ca

PLS-DA for Vis-NIR and SWIR (without wavelength selection)

The following supplement analysis explores the performance of PLS-DA models without wavelength selection for the Vis-NIR and SWIR wavelength ranges. Our goal was to evaluate the impact of not using wavelength selection on the model performance. The same pre-processing techniques and sample size were used as described in the main paper.

Table S1. PLS-DA results for mechanical damage classification using Vis-NIR wavelength range (without wavelength selection).

**Calibration**				
	True class			Recall (%)
Predicted class		Nil/low	Medium/high	
	Nil/low	43	4	87.80
	Medium/high	6	45	91.80
Precision (%)		91.80	87.80	
**Cross-validation**				
	True class			Recall (%)
Predicted class		Nil/low	Medium/high	
	Nil/low	43	5	87.80
	Medium/high	6	44	89.80
Precision (%)		89.80	87.80	
**Test**				
	True class			Recall (%)

Predicted class		Nil/low	Medium/high	
	Nil/low	17	1	81.00
	Medium/high	4	21	95.50
Precision (%)		95.50	81.00	

Table S2. PLS-DA results for mechanical damage classification using SWIR wavelength range (without wavelength selection).

**Calibration**				
	True class			Recall (%)
Predicted class		Nil/low	Medium/high	
	Nil/low	50	5	92.60
	Medium/high	4	41	89.10
Precision (%)		89.10	92.60	
**Cross-validation**				
	True class			Recall (%)
Predicted class		Nil/low	Medium/high	
	Nil/low	49	6	90.70
	Medium/high	5	40	87.00
Precision (%)		87.00	90.70	
**Test**				
	True class			Recall (%)
Predicted class		Nil/low	Medium/high	
	Nil/low	17	2	94.40
	Medium/high	1	24	92.30
Precision (%)		92.30	94.40	
